# NOTCH1: A Novel Player in the Molecular Crosstalk Underlying Articular Chondrocyte Protection by Oleuropein and Hydroxytyrosol

**DOI:** 10.3390/ijms24065830

**Published:** 2023-03-18

**Authors:** Veronica Panichi, Irene Bissoli, Stefania D’Adamo, Flavio Flamigni, Silvia Cetrullo, Rosa Maria Borzì

**Affiliations:** 1 Laboratorio di Immunoreumatologia e Rigenerazione Tissutale, Laboratorio di Patologia delle Infezioni Associate all’Impianto, IRCCS Istituto Ortopedico Rizzoli, 40136 Bologna, Italy; 2 Dipartimento di Scienze Biomediche e Neuromotorie, Università di Bologna, 40138 Bologna, Italy; 3 Laboratorio di Immunoreumatologia e Rigenerazione Tissutale, IRCCS Istituto Ortopedico Rizzoli, 40136 Bologna, Italy

**Keywords:** osteoarthritis, chondrocytes, LPS, oxidative stress, inflammation, NOTCH1, MMP-13, nutraceuticals

## Abstract

Osteoarthritis (OA) is the most common joint disease, but no effective and safe disease-modifying treatment is available. Risk factors such as age, sex, genetics, injuries and obesity can concur to the onset of the disease, variably triggering the loss of maturational arrest of chondrocytes further sustained by oxidative stress, inflammation and catabolism. Different types of nutraceuticals have been studied for their anti-oxidative and anti-inflammatory properties. Olive-derived polyphenols draw particular interest due to their ability to dampen the activation of pivotal signaling pathways in OA. Our study aims to investigate the effects of oleuropein (OE) and hydroxytyrosol (HT) in in vitro OA models and elucidate their possible effects on NOTCH1, a novel therapeutic target for OA. Chondrocytes were cultured and exposed to lipopolysaccharide (LPS). Detailed analysis was carried out about the OE/HT mitigating effects on the release of ROS (DCHF-DA), the increased gene expression of catabolic and inflammatory markers (real time RT-PCR), the release of MMP-13 (ELISA and Western blot) and the activation of underlying signaling pathways (Western blot). Our findings show that HT/OE efficiently attenuates LPS-induced effects by firstly reducing the activation of JNK and of the NOTCH1 pathway downstream. In conclusion, our study provides molecular bases supporting the dietary supplementation of olive-derived polyphenols to revert/delay the progression of OA.

## 1. Introduction

Despite different timing across species, healthy articular cartilage is a post-mitotic tissue committed to failure with age [1]. 

This “programmed” failure may be even anticipated by conditions that contribute to aging of the articular cartilage, such as low-grade systemic inflammation, obesity and trauma, both in humans and in animal models. Indeed, the correct architecture and functionality of articular cartilage are guaranteed by the active maintenance of the so-called “maturational arrest” that prevents chondrocytes entering their default route to hypertrophy and terminal differentiation, and subsequent endochondral ossification. This block is guaranteed by the correct functionality of cellular homeostatic mechanisms such as autophagy that ensures the disposal of damaged organelles and molecules, thus also counteracting the activation of signaling pathways that sustain osteoarthritis (OA) pathogenesis [2,3]. Therefore, the prevalence of human OA is gradually increasing, along with increased life span and the increasing impact of obesity and metabolic syndrome [4], which may impair the cellular homeostatic mechanisms [5]. A recent survey indicates that OA affects nearly 7% of the population worldwide [4] and this percentage is at least doubled in people with an age higher than 60, with women more affected than men [6]. Given the dramatic impact on both the quality of life of affected individuals and expenditure of the national health systems, much effort has been devoted to developing targeted therapies that may delay the occurrence and progression of OA. However, due to its multifactorial nature and heterogeneity [7,8], there have been no disease-modifying therapies available to date, and pharmacological treatment mainly addresses pain management. 

On the other hand, recent evidence has pointed at the usefulness of dietary intervention to counteract the progression of degenerative, non-communicable diseases linked to aging. A large European project (http://www.nu-age.eu (accessed on 21 September 2022)) listed at clinicaltrials.org has collected multiple findings showing that adherence to the Mediterranean diet even has the ability to rejuvenate the epigenetic fingerprinting of aging [9]. Olive oil is a basic component of the Mediterranean diet, and the efficacy of olive-derived polyphenols in the treatment of both human OA or surgically induced OA in animal models has already been underlined with multiple evidence “from bench to bedside” [10]. Olive-derived bioactive compounds, such as oleocanthal, oleuropein (OE), tyrosol, and hydroxytyrosol (HT), show interesting anti-inflammatory properties in many inflammatory or degenerative conditions. Recent “state of the art” reviews have been published providing molecular evidence whereby major olive oil polyphenols exert their protective effects on human health [3,11]. These nutraceuticals are both available from dietary intake or alternatively from nutritional supplements, with these molecules being easily obtained from oil waste products. 

In terms of concentration, the main polyphenols in olives are OE, its metabolite 2-(3,4-dihydroxyphenyl)-ethanol, known simply as HT, and α-tocopherol [12,13,14]. Detailed information about the biodistribution and metabolism of OE and HT is described elsewhere [15]. With a concentration up to 14% in the fruit, OE is the most abundant phenolic compound in olives, even though in the so-called “green phase” of fruit maturation, its concentration progressively decreases [16]. 

In the present study, we employed both primary chondrocytes as well as C28/I2 immortalized chondrocytes, widely used for their phenotype that more closely matches that of differentiated articular chondrocytes [17] compared to other available chondrocyte cell lines. C28/I2 chondrocytes were used to explore the signaling mechanisms, since the experimental settings required a high cell availability. 

To set up in vitro models, we exposed the cells to lipopolysaccharide (LPS) to mimic what happens in metabolic OA [18] as also confirmed in animal models [19], where a high-fat diet is responsible for an unhealthy microbiota that causes increased gut permeability and the absorption of LPS, with high serum levels. This seems to be a mechanism shared by many chronic degenerative diseases. An increased serum level of LPS is then able to activate innate immunity via triggering Toll-Like receptors 4 (TLR4) in macrophages and chondrocytes [20]. Noteworthily, increased expression of TLR4 has been described in OA [21].

Downstream of TLR4 engagement, several signaling pathways become activated, including the nuclear factor kappa-light-chain-enhancer of activated B cells (NF-κB), and the mitogen-activated protein kinases (MAPKs) p38 and c-Jun NH_2_-Terminal Kinase (JNK), the latter leading to the phosphorylation-mediated activation of c-Jun, a member of the Activator protein 1 (AP-1) family [22]. These events are finally responsible for the activated transcription of pro-inflammatory and catabolic molecules [20,23]. Among the latter, Matrix Metallopeptidase 13 (MMP-13) has been considered as a key protagonist in driving the failure of cartilage extracellular matrix beyond a no-return point [24,25]. MMP-13 is, therefore, a candidate target for therapeutic strategies. Mengshol et al. previously reported the requirement of an interaction between Runt-related transcription factor 2 (*RUNX2*) and AP-1 in MMP-13 transcription [26]. Recent work from our and other groups has underlined that, in MMP-13 transcription, a fundamental role is also played by Notch homolog 1 (NOTCH1) signaling that promotes RUNX2 activation [27,28]. Since a crosstalk between JNK and the NOTCH1 signaling pathway has been described in macrophages in the regulation of inflammatory responses, with the former activating the latter [29], we explored whether this crosstalk also occurs in articular chondrocytes. In addition, we investigated whether OE and HT are able to attenuate JNK activation and the LPS-induced transcription of inflammatory and catabolic genes.

We found that pretreatment with either OE or HT significantly dampened LPS-induced expression of most inflammatory and catabolic genes. This effect was achieved by means of the reduction in LPS-induced ROS and, consequently, of the activation of major signaling pathways: first JNK, and then NOTCH1. 

## 2. Results

### 2.1. LPS Induces the Transcription of Inflammatory Genes in Both Primary Chondrocytes and C-28/I2 Cells 

**Figure 1:**
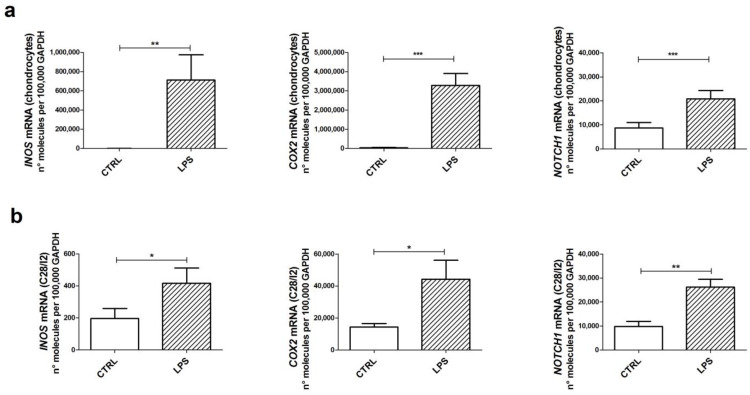
Effects of lipopolysaccharide (LPS) on inflammatory responses. mRNA expression of inflammation markers in primary chondrocytes (**a**) and C28/I2 chondrocytes (**b**) significantly increased 6 h after LPS stimulation. Data from cells treated with LPS (dashed pattern) were compared to appropriate control and obtained from the same set of samples (*n* = 13 to 15 different primary chondrocyte cultures or *n* = 5 for C28/I2). Quantification of expression levels was calculated for each target gene and normalized using the reference housekeeping gene GAPDH. Values obtained in chondrocytes were calculated according to the formula 2^−ΔCt^ and expressed as number of molecules per 100,000 GAPDH molecules. Results are means ± SEM. Statistical analysis was performed by Student’s *t* test for paired samples (* *p* < 0.05; ** *p* < 0.01; and *** *p* < 0.001).

We firstly assessed the suitability of C28/I2 cells as a convenient cell model to tease out the molecular crosstalk downstream exposure to LPS. To this end, we compared primary articular chondrocytes and C28/I2 cells, both exposed to LPS for 6 h (Figure 1). 

We investigated the induction of genes contributing to the amplification of the inflammatory/hypertrophic deregulation of chondrocytes: Inducible Nitric Oxide Synthase (*INOS*), involved in the generation of nitric oxide species and exerting a key role in OA pathogenesis [30]; Cyclooxygenase-2 (*COX2*), involved in the production of prostaglandins, mediators of pain [31]; and *NOTCH1*, which we recently showed as pivotal in supporting many pathways of loss of maturational arrest in chondrocytes [27]. 

With both cell types, we found a statistically significant increase in the induction of these target genes, although C28/I2 cells were somehow less reactive than primary chondrocytes [17]. 

### 2.2. OE and HT Show Antioxidant Activities against LPS-Induced ROS Production in C28/I2 Cells

**Figure 2:**
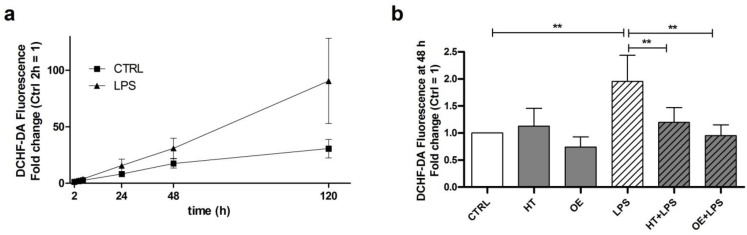
Antioxidant effect of OE and HT in C28/I2 chondrocytes. (**a**) LPS treatment increases ROS production progressively from time 2 h to 120 h compared to control. ROS were assessed by fluorescence intensity of DCHF-DA. (**b**) Pretreatment of C28/I2 chondrocytes with olive-derived nutraceuticals OE and HT showed a statistically significant reduction in LPS-induced oxidative stress at 48 h. Data represent results from multiple analysis (*n* = 4) of quadruplicate samples for each experimental condition expressed as mean ± SEM and normalized with respect to the control at 48 h. Statistical analysis was performed by ANOVA, followed by Newman–Keuls’ post hoc test, with ** *p* < 0.01.

As previously reported [32], LPS exposure of chondrocytes yields the production of intracellular ROS as assessed by means of 2′-7′-dichlorofluorescein diacetate (DCHF-DA), a fluorogenic dye that allows a sensitive quantification of ROS within the cells. A time-course assessment of ROS levels in C28/I2 cells after exposure to LPS is shown in Figure 2a. LPS treatment provoked a progressive increase in ROS production starting from 2 h up to 120 h. ROS induction following LPS exposure was comparable with that elicited by exposure to two positive controls, hydrogen peroxide (100 µM) and its stabilized form tert-butyl hydrogen peroxide (TBHP, 55 µM). We found that the longer the incubation with LPS, the larger the increase in ROS production (Figure 2a). Anyway, due to the high variability at 120 h post-stimulation, the 48 h time point was chosen for statistical analysis. We, therefore, confirmed even in C28/I2 chondrocytes that LPS induces a significant increase in ROS production (Figure 2b). Noteworthily, in these experimental settings, we observed the antioxidant properties of HT and OE. Indeed, C28/I2 cells pretreated for 16 h with either one of the two polyphenols prior of exposure to LPS showed a statistically significant decrease in LPS-induced ROS production. These results confirm that OE and HT exert an antioxidant action and a consequent protective effect inhibiting oxidative stress in our experimental model. 

### 2.3. OE and HT Prevent LPS-Mediated Induction of Inflammatory and Catabolic Genes following LPS Exposure in Chondrocytes

**Figure 3:**
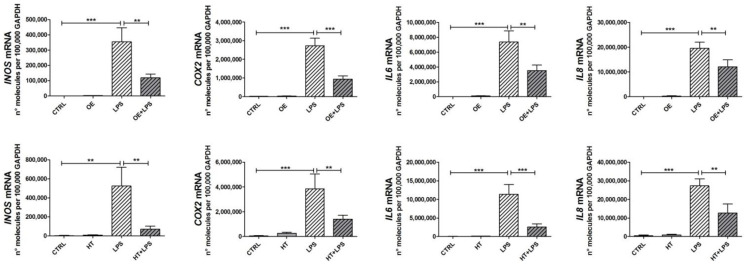
Effects of OE and HT on inflammatory markers. LPS treatment increases markers of OA in primary OA chondrocytes and pretreatment with either OE (**upper graphs**) or HT (**lower graphs**) efficiently inhibits this effect. Results are means ± SEM (*n* = 5 to 9 different primary chondrocyte cultures), normalized using the reference housekeeping gene GAPDH according to the formula 2^−ΔCt^ and expressed as number of molecules per 100,000 GAPDH molecules. Statistical analysis was performed by ANOVA, followed by Newman–Keuls’ post hoc test, with ** *p* < 0.01, *** *p* < 0.001.

**Figure 4:**
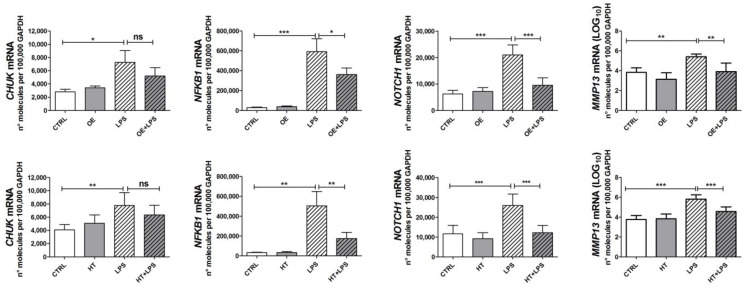
Effects of OE and HT on relevant intermediates of signaling pathways, and on the pivotal catabolic enzyme in OA. LPS treatment increases the expression of these genes in primary OA chondrocytes and pretreatment with either OE (**upper row**) or HT (**lower row**) efficiently inhibits this effect. Results are means ± SEM (*n* = 4 to 9 different primary chondrocyte cultures), normalized using the reference housekeeping gene GAPDH according to the formula 2^−ΔCt^ and expressed as number of molecules per 100,000 GAPDH molecules. Statistical analysis was performed by ANOVA, followed by Newman–Keuls’ post hoc test, with * *p* < 0.05, ** *p* < 0.01, *** *p* < 0.001. ns = not significant.

ROS have been recognized to activate the signaling pathways downstream of LPS exposure [20], i.e., NF-κB [33] and the MAPK pathways [34]. Since both OE and HT were able to reduce the ROS increase following LPS stimulation, we speculated that this could be reflected in a reduced activation of signaling pathways. Therefore, we investigated OE/HT’s ability to reduce the expression of a wide panel of genes. These included inflammatory genes, such as *INOS*, *COX2*, *IL6* and *IL8* (Figure 3), as well as other genes coding for relevant intermediates of signaling pathways, such as *NFKB1* (indicating activation of the NF-κB) [33], *NOTCH1* (responsible for the hypertrophy of chondrocytes in close connection with NF-κB) [35], *CHUK* (IKKα, a downstream target of the NOTCH1 pathway itself [36], with relevant roles in the NF-κB pathway and chondrocyte differentiation [37]) (Figure 4). We also assessed the effects of OE and HT in mitigating the increased expression of *MMP13* (Figure 4). Pretreatment with the nutraceuticals significantly attenuated the LPS-dependent increased expression of inflammatory (*INOS*, *COX2*, *IL6* and *IL8*) and catabolic (*MMP13*) genes as well as of *NFKB1* and *NOTCH1*, suggesting the inhibition of both NF-κB and the NOTCH1 pathways. 

### 2.4. OE and HT Prevent JNK Activation following LPS Exposure in Chondrocytes 

**Figure 5:**
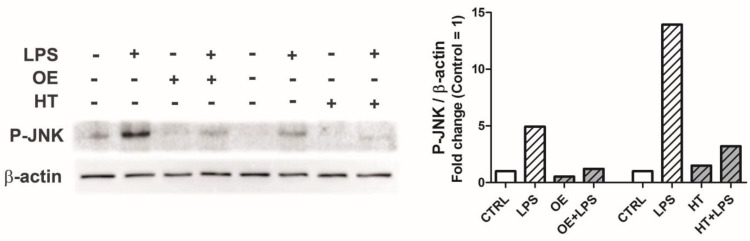
**Left**: activation of JNK by LPS exposure in primary OA chondrocytes. To account for the different solubility requirements of the two polyphenols, two different set of samples were prepared, with either DMSO as a vehicle (to assess OE effects) or ethanol (to assess HT effects). **Right graph**: quantification of phosphorylated JNK intensity relative to β-actin, and expressed as fold change relative to the control for each set of samples. Stimulation with LPS increased the levels of phosphorylated JNK while pretreatment with either OE or HT prevented this activation.

As shown in Figure 5, pretreatment with OE/HT proved able to reduce JNK activation (P-JNK) in primary chondrocytes as assessed with Western blotting.

### 2.5. Following LPS Exposure, JNK Activation Triggers NOTCH1 Pathway Leading to Increased MMP-13 Gene Transcription

**Figure 6:**
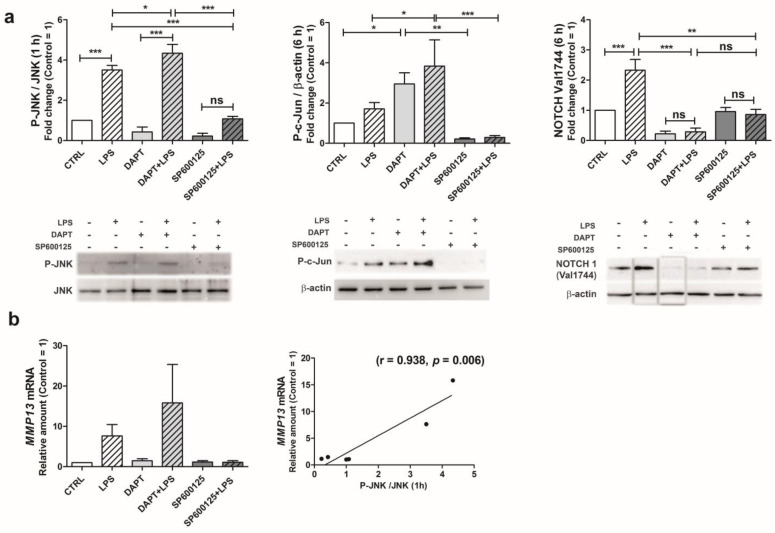
Activation of JNK pathway (phosphorylation of JNK and c-Jun) and of NOTCH1 (cleavage of Val1744) following LPS exposure and their effects on MMP-13 transcription. (**a**) In C28/I2, stimulation with LPS (1 h) strongly increased the levels of phosphorylated JNK and, after 6 h, that of cleaved NOTCH1. DAPT-mediated inhibition of NOTCH1 cleavage (Val1744) and activation enhances phosphorylated JNK after LPS exposure. Representative Western blots are shown for P-JNK (*n* = 4), P-c-Jun (*n* = 7) and NOTCH1 Val1744 (*n* = 3) In the latter case, rearrangement (switch) of LPS and DAPT lanes was performed for consistency with the other Western blots (see Supporting information file). (**b**) MMP-13 mRNA (6 h) showed a trend that was found in correlation with that of phosphorylated JNK (1 h). Statistical analysis was performed by ANOVA, followed by Newman-Keuls’ post hoc test, with * *p* < 0.05, ** *p* < 0.01, *** *p* < 0.001. ns = not significant.

The crosstalk between JNK and NOTCH1 pathway was investigated in C28/I2 chondrocytes, exploiting conventional inhibitors. At 1 h posttreatment, LPS was able to strongly increase the level of phosphorylated JNK (Figure 6a, left). As expected, cells treated with the JNK inhibitor SP600/125 showed a significantly decreased JNK activation in LPS-stimulated conditions. Instead, the DAPT-mediated inhibition of canonical NOTCH pathway activation further enhanced the level of activated JNK. In addition, pretreatment with DAPT significantly increased the degree of c-Jun phosphorylation (6 h) in both control and LPS-stimulated conditions (Figure 6a, middle). These results are in keeping with already-reported inhibitory activity exerted by NOTCH1 on JNK activation [38]. Figure 6a (right) also illustrates how the levels of activated NOTCH (cleaved at Val1744) showed a highly significant increase at 6 h post LPS treatment. As expected, DAPT significantly abrogated the formation of cleaved NOTCH in LPS-stimulated conditions. Treatment with SP600/125 instead resulted in levels similar to those of control samples, in both the control and stimulated conditions, confirming the involvement of JNK in modulating NOTCH signaling pathway in our experimental model. 

Indeed, the assessment of MMP-13 mRNA level in C28/I2 cells (6 h after LPS) showed a trend that confirmed the pivotal role of JNK, and was suggestive of the contribution of this pathway for MMP-13 expression (Figure 6b).

Collectively, these data suggest that in MMP-13 induction, phosphorylated JNK acts by both enhancing NOTCH1 activation [29] and mediating the phosphorylation of c-Jun for MMP-13 induction. On the other hand, NOTCH1 has a mitigating effect on JNK activation. Indeed, the highest levels of P-c-Jun were observed after DAPT treatment in both basal and LPS-stimulated conditions. In keeping with this observation, after LPS treatment, P-c-Jun showed higher values in chondrocytes treated with NOTCH siRNA compared to control siRNA (Supplementary Figure S1).

### 2.6. OE/HT Pretreatment of Both C28/I2 and Primary Chondrocytes Inhibits MMP-13 Synthesis and Release following LPS Exposure

Collectively, the above-reported data describe the signaling network leading to MMP-13 activation, and in particular, the central role of JNK that we found inhibited by OE/HT pretreatment. Therefore, we then aimed at investigating whether OE and HT delivered to C28/I2 or primary chondrocytes were able to dampen the synthesis and/or the release of MMP-13. 

**Figure 7:**
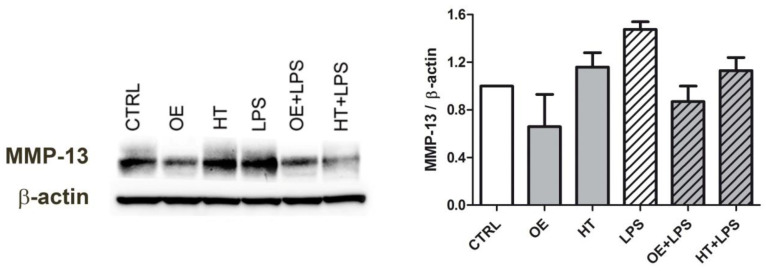
Inhibiting effects of OE and HT on LPS-induced MMP-13 expression in C28/I2 chondrocytes, following 6 h exposure to LPS. Right graph: quantification of MMP-13 intensity relative to β-actin (2 experiments).

C28/I2 cells pretreated (16 h) with either OE or HT and then evaluated at 6 h following LPS exposure showed reduced MMP-13 intracellular levels (Figure 7).

Interestingly, differently from OE, exposure to plain HT itself led to an increased MMP-13 expression in C28/I2 chondrocytes. However, HT pretreatment was successful in reducing LPS-dependent MMP-13 induction. 

However, MMP-13 levels are more conveniently assessed in the supernatant, since this collagenase is not normally expressed in healthy chondrocytes nor stored intracellularly, but promptly induced upon stimulation and released. In addition, this marker is better assessed in primary chondrocytes that are endowed with relevant genes for ECM anabolism and catabolism [17].

Therefore, we moved to assess the inhibitory effects of pretreatment with either OE or HT on the LPS-induced release of MMP-13 on the supernatants of primary chondrocytes, seeded at high density to reproduce the differentiated phenotype of chondrocytes within the tissue [39]. Three different primary cultures were tested, and each condition was tested in triplicate wells. 

**Figure 8:**
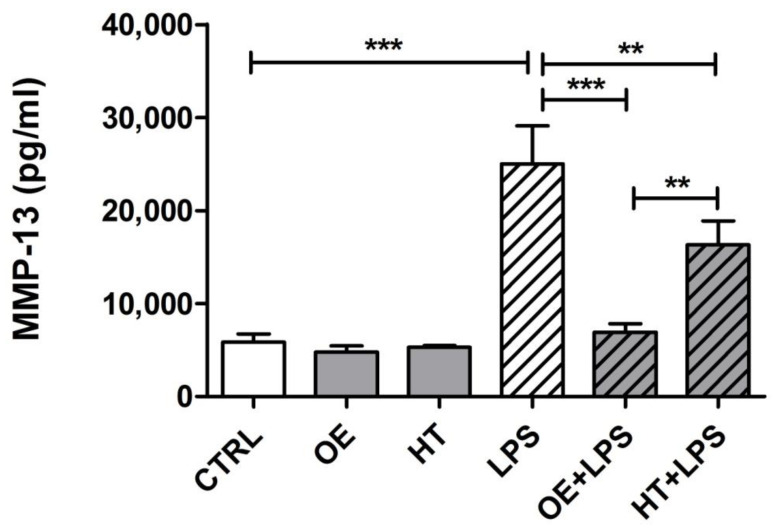
Inhibiting effects of OE and HT pretreatment on LPS-induced MMP-13 release from OA chondrocytes, following 24 h exposure. Cumulative data obtained from three patients (mean ± SEM, *n* = 3), with triplicate wells seeded for each condition for each patient. Oleuropein was confirmed as exhibiting a complete inhibiting activity on MMP-13 release induced by LPS treatment. Data were compared by ANOVA, followed by Newman–Keuls’, with ** *p* < 0.01, *** *p* < 0.001.

The release was assessed after a 24 h exposure to the inflammatory stimulus, as previously reported [40]. The results obtained (Figure 8) showed that OE exhibited the more effective protecting activity against LPS, since the decrease was statistically significant with *p* < 0.001. Supplementary Figure S2 shows the data obtained from each primary culture. The three different cultures exhibited a similar pattern. In addition, the levels of MMP-13 in OE + LPS samples were always significantly lower compared to those with HT + LPS treatment and no statistically significant differences were observed between MMP-13 levels of OE + LPS compared to control samples. 

## 3. Discussion

In this study, we used an experimental setting useful to mimic in vitro the inflammatory environment of OA and selected LPS as a stimulus to replicate the condition of metabolic OA [18]. We explored the mitigating effects of OE and HT on MMP-13 release and upstream signaling pathways. Primary OA chondrocytes were tested at high density, a condition that promotes a differentiated phenotype [39], useful to draw meaningful “translational” information, to be transferred from bench to bedside. 

The LPS triggering of TLR4 induces ROS generation via multiple mechanisms. This is a relevant observation, since, as also stated elsewhere, “excess levels of these ROS not only cause oxidative-damage but, perhaps more importantly, cause a disruption in cell signaling pathways that are redox-regulated, including Akt and MAP kinase signaling [41]”. Most of the knowledge accumulated so far derives from studies performed on macrophages. However, many shared features have been described for OA chondrocytes and activated macrophages [42], including the ability to produce reactive oxygen species. Most of the early ROS generation after the triggering of TLR4 derives from a mechanism that involves the IRAK1/ERK activation of a dimer composed of p67phox (a 67 kD subunit of the multiprotein NADPH oxidase, responsible for a burst of superoxide) and NOX2 [43,44]. This ROS burst and the downstream activation of inflammatory pathways are indeed prevented by either TLR4 inhibition, NADPH oxidase inhibition or N-acetylcysteine ROS scavenging activity [44]. However, a nutraceutical-based strategy devoted to counteracting excessive ROS generation is a convenient approach whereby maintaining cell homeostasis, rather than TLR inhibition. Indeed, TLR4 has been recently shown to act as a double edge sword in OA progression, since it is responsible for both activation of inflammatory signaling, but at the same time for induction of homeostatic pathways, leading to antioxidant activities (SOD2), repair mechanisms (OGG1, responsible for mitochondrial functionality) and autophagy induction [45]. 

Despite ROS may exert both detrimental and beneficial effects on longevity, depending on condition and species [46], accumulating evidence indicates that in the case of OA, ROS may trigger a lot of pathogenic pathways in chondrocytes sustaining DNA damage, senescence, inflammation and ECM remodeling [5,41]. Therefore, it is noteworthy that either OE or HT significantly prevented LPS-dependent ROS increase in C28/I2 cells after prolonged exposure to LPS. To our knowledge, this work, for the first time, points at an antioxidant activity of olive-derived polyphenols in chondrocytes exposed to LPS. 

As expected, this antioxidant activity reduced the activation of signaling pathways. ROS may indeed activate NF-κB [33] and also the MAPKs [34,41]. This resulted in the increased expression of inflammatory markers (*INOS*, *COX2*, *IL6*, and *IL8*), but also of relevant intermediates in signaling pathways, such as *NOTCH1* and *CHUK*, a target gene of the NOTCH1 pathway [36]. The latter has been previously found to be activated in macrophages following their exposure to LPS [47]. NOTCH has been connected with aging [36] and indeed, in epithelial cells, its transcriptional regulation is controlled by p53 [48], a critical effector of aging. In addition, in articular cartilage *NOTCH1* is epigenetically controlled by miR146a that counteracts aging by inhibiting *NOTCH1*, *IL6* and *IL8* [49]. 

Moreover, relevant activity of LPS was also observed in the induction of *NFKB1*, a marker of NF-κB activation [33]. LPS induced the expression of *MMP13*, a gene that is dispensable in healthy cartilage metabolism, but that, once re-expressed in OA cartilage, indicates the triggering of hypertrophy towards terminal differentiation [24,25]. Being a gene not normally expressed in chondrocytes, *MMP13* level was found to be quite variable among the different primary cultures enrolled, in keeping with the notion of “high” and “low” responders among OA patients [50], in opposite correlation with the basal level. Indeed, the extent of MMP-13 production following exposure to growth factors and cytokines has been shown to be a function of the physiologic state of the cells [50]. 

In conclusion, LPS exposure significantly up-regulated the expression of inflammatory markers and relevant players in OA pathophysiology, and these effects were significantly dampened by pretreatment with either OE or HT in keeping with other literature reports [3,10,11,51]. 

Among the signaling pathways that ROS can activate downstream of LPS exposure [20], we selected JNK and investigated whether OE and HT were able to prevent its activation. JNK indeed could be critical for its ability of regulating NOTCH1 activation [29], and therefore its downstream target MMP-13 [28]. The effects of modulating the signaling pathways upstream *MMP13* were investigated in C28/I2 cells, a convenient model whereby exploring signaling mechanisms [52]. However, these cells do not fully substitute primary chondrocytes with reference to the expression of extracellular matrix anabolic and catabolic genes [17]. 

Most of the novelty of our work lies in the effects of OE and HT on the axis JNK > NOTCH1. We chose to focus on the JNK pathway, in view of its reported ability to activate the NOTCH1 pathway [29]. In addition, c-Jun, involved in *MMP13* transcription, in association with RUNX2 [26], is a substrate of JNK activity, and the “phosphorylation of Jun by JNK is a prerequisite for the ability of the AP-1 complex to execute transcriptional activation [53]”. Previous studies showed a peak in JNK activation 1 h following exposure to the inflammatory stimulus [53], despite a certain variability occurring in primary chondrocyte cultures in the degree of response and kinetics. Furthermore, the same report suggested a more central role of JNK in inflammatory responses compared to the other MAPKs [53]. Our findings show that both OE and HT were able to prevent the phosphorylation-dependent activation of JNK in primary chondrocytes. Further investigation carried out on C28/I2 chondrocytes shows that JNK inhibition leads to reduced NOTCH1 activation, while, conversely, NOTCH1 pathway inhibition via DAPT treatment tends to increase the level of phosphorylated JNK and of its downstream signaling intermediate phosphorylated c-Jun, disclosing the occurrence of a negative feedback exerted by NOTCH on JNK. This appeared confirmed by a higher level of phosphorylated c-Jun in chondrocytes treated with NOTCH1 siRNA. The findings of DAPT effects on JNK > P-c-Jun axis are in keeping with other literature reports that show that “Notch interferes with the scaffold function of JNK-interacting protein 1 to inhibit the JNK signaling pathway” [38]. Noteworthily, our present data together with previously published results [27,28] would suggest that, in chondrocytes, MMP-13 transcription depends on the combined activity of activated NOTCH1 (cleaved at Val1744, by γ secretase) and phosphorylated c-Jun. In addition, a crosstalk between Notch and NF-κB (noncanonical) signaling pathways has been described as enhancing the transcription of many OA relevant genes, including *IL6* and *MMP13* [35]. 

Previously, Gualillo and colleagues demonstrated a chondroprotective action of oleocanthal, another phenolic compound derived from olives, in a similar experimental setting [54,55], but to our knowledge, our study is the first that describes these effects for HT and OE in both primary chondrocytes and C28/I2 cells.

Based on our findings, OE pretreatment generally exerts the more penetrant activity in counteracting the effects of LPS exposure: ROS production, activation of signaling pathways and transcription of inflammatory/catabolic markers. This is in keeping with its already-reported ability of alleviating OA in both animal [56] and human, improving joint functional capacity in older people with high knee joint pain [57]. In addition, OE has been found to act as a senolytic/senomorphic agent promoting a pro-regenerative environment in chondrocytes, synovial and bone cells [58]. 

In conclusion, our study confirms a key role for NOTCH1 in the pathogenesis of OA, thus pointing at this signaling pathway as a potential target for therapy. However, as extensively reviewed elsewhere [27], NOTCH1 is a kind of double-edged sword with both homeostatic and unwanted effects. Therefore, to avoid the latter, attempts should be made to develop a strategy for preventing excessive activation while sparing its ability to inhibit JNK activation. From this perspective, the findings of our study, indicating that olive-derived polyphenols have remarkable chondroprotective effects and are able to modulate JNK and NOTCH1 activation, strongly support the urgency to investigate in vivo this chondroprotective action, in order to consider these compounds as a therapeutic option for OA management.

## 4. Materials and Methods

### 4.1. Cells Isolation and Treatment

For this study, two in vitro models were used: primary chondrocytes and C28/I2, an immortalized human chondrocyte line [17]. Human primary chondrocytes (*n* = 22) were isolated from knee cartilage derived from OA patients undergoing arthroplasty as previously indicated [27]. The study was conducted according to the guidelines of the Declaration of Helsinki and approved by the Ethics Committee of Istituto Ortopedico Rizzoli (ethic approval code: 0019715, approved on 28 September 2016), including documentation of written patient informed consent. Primary chondrocytes retrieval and expansion were conducted as described in [27], i.e., only P_0_ chondrocytes (i.e., cells that did not undergo subculturing and, therefore, retained proper chondrocyte differentiation) were used for functional studies. Then, chondrocytes were seeded at high density (62,500 cells/cm^2^) in 12-well plates. Under these conditions, chondrocytes are morphologically differentiated with a round-to-polygonal shape instead of the elongated shape of dedifferentiated chondrocytes [59]. In addition, they retain the expression of collagen 2, the major phenotypical marker of differentiated chondrocytes (Supplementary Figure S3). At a time of 48–72 h after seeding, cells were starved for 24 h before pretreatment with either OE or HT. C28/I2 chondrocytes were expanded and seeded with DMEM with 10% FBS at the same cell density. No starvation was performed with immortalized chondrocytes, since it has been previously shown to induce mitochondria damage or dysfunction [60]. Then, both cellular models were pretreated for 16 h with olive-derived nutraceuticals: 100 μM Oleuropein (OE, 92167, Sigma) and 100 μM Hydroxytyrosol (HT, 70604, Cayman chemical, MI, USA) before stimulation with 10 μg/mL lipopolysaccharide (LPS, L-2654, Sigma) for 1–120 h, depending on subsequent use, as described below. As an inhibitor of NOTCH1 cleavage at Val1744, the γ-secretase complex inhibitor DAPT (D5942, Sigma) was used. A stock concentration of 5 mM was prepared in DMSO and diluted to a final concentration of 5 µM in culture medium. SP600125 (SP600125, Tocris Bioscience, Bristol, UK) was 10 mM in DMSO and used at a final concentration of 10 µM.

### 4.2. Western Blot

Western blotting was carried out to evaluate the modulatory effects of nutraceuticals in the activation of signaling pathways downstream LPS exposure. To this end, cells were recovered by scraping the cells at 1 h or 6 h post-stimulation with a small volume of cold buffer. Pellets of either primary chondrocytes or C28/I2 were resuspended in RIPA Buffer (ThermoFisher, Waltham, MA, USA, 89900) and added with Halt™ Protease and Phosphatase inhibitor cocktail and EDTA 100x (78440, Thermo Fisher) according to the manufacturer’s instructions. Extraction was carried out via sonication then the samples were centrifuged for 15′ at 11,000× *g*. Total proteins were quantified by means of a Bradford Assay with Coomassie Brilliant Blue G-250 (AppliChem, Council Bluffs, IA, USA, A3480) dye. An equal amount of protein (in most cases 30 µg) was run in each experiment. SDS-PAGE was performed on a BioRad apparatus on 10–12% Acrylamide gels. Page RulerTM Plus Prestained Protein Ladder (26619, Thermo Fisher) was used as a molecular weight marker. Proteins were transferred on nitrocellulose or PVDF membranes, treated for the blocking of nonspecific bindings (5% dry milk in 0.1% Tween 20 in TBS), and then incubated overnight at 4 °C in agitation with the following primary antibodies: cleaved Notch1 (Val1744) (rabbit monoclonal anti-human NOTCH-1, clone D3B8, #4147, Cell Signaling Technology, 1:1000); phospho-JNK (rabbit polyclonal anti-Phospho-SAPK/JNK) (Thr183/Tyr185, #9251, Cell Signaling Technology, 1:1000); total SAPK/JNK (rabbit polyclonal, #9252, Cell Signaling Technology, 1:1000), Phospho-c-Jun (Ser73) (rabbit polyclonal, #9164, Cell Signaling Technology, 1:1000); total-c-Jun (t-cJun, rabbit polyclonal, #9162, Cell Signaling Technology, 1:1000); MMP-13 (goat polyclonal, AF511, R&D, 1:5000); and β-actin (mouse monoclonal, #A5316, Sigma-Aldrich, 1:5000). 

After three repeated washes in TSB + 0.05% Tween, the membranes were incubated 1 h at R.T. in 3% dry milk added with either anti-mouse, anti-rabbit or anti-goat secondary antibody (1:2000) (horseradish-peroxidase-conjugated anti-mouse IgG, 70765, Cell Signaling Technology; horseradish-peroxidase-conjugated anti-rabbit IgG, 70745, Cell Signaling Technology; or horseradish-peroxidase-conjugated anti-goat IgG, Jackson ImmunoResearch). Western blot bands were detected using ECL Select (GE Healthcare) with a ChemiDoc MP system (BioRad). β-actin (A5316, Sigma) served as the loading control and for the normalization of band intensity quantification carried out with the data analysis software Image Lab (Version 4.1.). For the comparison shown in Figure 6, the following analyses were undertaken: P-JNK: P-JNK values normalized to the corresponding levels of total JNK (in this analysis, the 54 kDa JNK2 form was considered that appeared to be the prevalent form in total JNK Western blot); P-c-Jun: phosphorylated c-Jun values normalized to the level of β-actin; and cleaved NOTCH1 (Val1744): cleaved NOTCH1 values normalized to β-actin. 

### 4.3. DCHF-DA Cellular ROS Assay

C28/I2 chondrocytes were seeded at a density of ~15,000 cells/cm^2^ in a 96-well plate with a clear flat bottom (Sarstedt). At a time of 24 h later, a 16 h pretreatment with either 100 μM OE or 100 µM HT was performed. Afterwards, DCHF-DA (2′,7′-dichlorofluorescein diacetate) was added following the manufacturer’s instructions for a Cellular ROS detection assay kit (ab113851, Abcam, Waltham, MA, USA). Cells were washed once with 100 μL 1X Dilution Buffer and then incubated for 30′ at 37 °C with 25 μM DCHF-DA in 1X Buffer. The solution was then removed and after being washed, the cells were treated with 10 μg/mL LPS. A DCHF-DA signal was detected at 2–4–6–24–48–120 h at Ex/Em = 485/535 nm with an Infinite NANO M+ plate reader (Tecan). Amounts of 55 μM Tert-Butyl Hydrogen Peroxide (TBHP, Abcam) and 100 μM hydrogen peroxide were used as positive controls. Each experimental condition was tested in triplicate or quadruplicate; the experiment was repeated five times.

### 4.4. Real-Time RT-PCR Analysis

Total RNA was extracted with Trizol (15596-026, Invitrogen, Waltham, MA, USA) from cell pellets collected 6 h after stimulation with LPS, according to the manufacturer’s instructions. Total RNA was reverse-transcribed using SuperScript VILO cDNA Synthesis Kit (11754-050, Invitrogen) following the manufacturer’s protocol. Real-time RT PCR analysis was performed employing TB Green^®^ Premix Ex Taq™ II (Tli RNase H Plus) (RR82LR, TaKaRa) and following the standard protocol: Takara *Ex Taq* Hot Start DNA Polymerase activation 95° followed by 45 cycles (denaturation 95° and amplification with an annealing temperature variable according to the primer design as indicated in Table 1). mRNA quantification was calculated for each target gene and normalized using GAPDH as a reference gene. The results obtained from both primary chondrocytes and C28/I2 were expressed according to the formula 2^−ΔCt^ and represented as number of molecules per 100,000 GAPDH molecules. Primer specificity was assessed by the software Primer-BLAST, and further checked via the evaluation of melting curves. The sequences of selected primer pairs for each target genes and annealing temperature are shown below (Table 1).

### 4.5. ELISA Assay for MMP-13 Quantification

Primary chondrocytes were seeded and treated as described in 4.1. After 24 h of LPS exposure, the cell supernatants were collected and preserved at −20 °C until use. Human total MMP-13 was detected using the DuoSet ELISA Development System (DY511, R&D). Then, 96-well microplates were coated O.N. at R.T. with mouse anti-human MMP-13 Capture antibody (a final concentration of 4µg/mL) following the manufacturer’s instructions. After the incubation, the solution was aspirated and each well was washed 4 times with wash buffer (0.05% Tween 20 in PBS). This step was repeated after each incubation. The supernatant samples were appropriately (1:15) diluted in reagent diluent (1% BSA in PBS); 100 µL was added to each well and incubated at R.T. for 2 h, and repaired from light. For each experimental condition, samples were tested in triplicates. Then, the detection step was started with Human MMP-13 Biotinylated Goat anti-human MMP13 Detecting Antibody (R&D) diluted in reagent diluent (final concentration: 100 ng/mL) and left at R.T. for 2 h. Subsequently, 100 µL of working dilution of streptavidin-HRP (1:200 from stock solution) was added to each well and incubated at R.T. for 20′. The detection of bound MMP-13 was achieved by adding 100 µL of the substrate solution consisting of a 1:1 mixture of color reagent A (H_2_O_2_) and color reagent B (tetramethylbenzidine) (R&D Systems, DY999); after 20′, the reaction was blocked by adding 100 µL of stop solution (2 N H_2_SO_4_; R&D Systems, DY994). The optical density of each well was determined immediately at the end of incubation using the Infinite NANO M+ plate reader (Tecan) set at 450 nm with a wavelength correction set at 540 nm. The value of each sample was analyzed referring to a seven-point standard curve determined with recombinant human MMP-13 Standard (R&D Systems, #843400, stock concentration 250 ng/mL, and used at a two-fold dilution from 4000 to 62.5 pg/ml) according to the manufacturer’s protocol. 

### 4.6. Statistical Analysis

Data are represented as the mean ± standard error of mean (SEM) and compared by mean of Student’s *t*-test for paired samples or ANOVA (with Newman–Keuls’ post hoc test) where appropriate using the GraphPad Prism 5.0 software (GRAPHPAD SOFTWARE, La Jolla, CA, USA). In the case of MMP-13, given the high variability in the level of gene expression in the different primary cultures evaluated, the comparison was performed after variance normalization by using the Log_10_ of the values. In all the figures, in both the main manuscript and the supplementary material, a consistent way of representing the conditions was used, as follows: no pattern: unstimulated samples; dashed pattern: LPS-stimulated samples; white fill: no pretreatment; grey fill: pretreatment with modulators of signaling (nutraceutical/DAPT/SP600125). Differences were considered significant when *p* < 0.05 with * *p* < 0.05; ** *p* < 0.01; and *** *p* < 0.001.

## Figures and Tables

**Table 1:** List of primers used for Real time RT-PCR.

Gene	Forward Primer	Reverse Primer	Amplicon Size (Annealing T)
*GAPDH*	CGGAGTCAACGGATTTGG	CCTGGAAGATGGTGATGG	**218 bp (60 °C)**
*CHUK(IKKα)*	GCACAGAGATGGTGAAAATCATTG	CAACTTGCTCAAATGACCAAACAG	**86 bp (60 °C)**
*NFKB1*	CAGGAGACGTGAAGATGCTG	AGTTGAGAATGAAGGTGGATGA	**109 bp (60 °C)**
*NOTCH1*	CCTGAAGAACGGGGCTAACA	GATGTCCCGGTTGGCAAAGT	**127 bp (60 °C)**
*MMP13*	TCACGATGGCATTGCT	GCCGGTGTAGGTGTAGA	**277 bp (58 °C)**
*INOS*	ACATTGATCAGAAGCTGTCCCAC	AAAGGCTGTGAGTCCTGCAC	**235 bp (58 °C)**
*COX2*	CAGCACTTCACGCATCAGTTT	GCGCAGTTTACGCTGTCTA	**129 bp (58 °C)**
*IL6*	TAGTGAGGAACAAGCCAGAG	GCGCAGAATGAGATGAGTTG	**184 bp (60 °C)**
*IL8*	CCAAACCTTTCCACCC	ACTTCTCCACAACCCT	**153 bp (60 °C)**

## Data Availability

Data reported in the study are available upon request to the corresponding author.

## Supplementary Materials

The following supporting information can be downloaded at: https://www.mdpi.com/article/10.3390/ijms24065830/s1.
