# Supplementary material for: NOTCH1: A Novel Player in the Molecular Crosstalk Underlying Articular Chondrocyte Protection by Oleuropein and Hydroxytyrosol
Source: Int J Mol Sci. 2023 Mar 18;24(6):5830. doi: 10.3390/ijms24065830 (PMC10058228; doi:10.3390/ijms24065830)
Supplement: Supplementary file 1 [file ijms-24-05830-s001.zip › ijms-2101755-supplementary.pdf]

SUPPLEMENTARY MATERIAL

NOTCH1: a novel player in the molecular crosstalk underlying articular chondrocyte protection by oleuropein and hydroxytyrosol

Veronica Panichi[1,], Irene Bissoli[2], Stefania D'Adamo[2], Flavio Flamigni[2], Silvia Cetrullo[2 #],and Rosa Maria Borzì[3 # *]

1 Laboratorio di Immunoreumatologia e Rigenerazione Tissutale, Laboratorio di Patologia delle Infezioni Associate all'Impianto, IRCCS Istituto Ortopedico Rizzoli, 40136 Bologna, Italy
2 Dipartimento di Scienze Biomediche e Neuromotorie, Università di Bologna, 40138 Bologna, Italy
3 Laboratorio di Immunoreumatologia e Rigenerazione Tissutale, IRCCS Istituto Ortopedico Rizzoli, 40136 Bologna, Italy
* Correspondence: rosamaria.borzi@ior.it; tel +39-051-6366803
#Equal contributors

Supplementary Figure S1

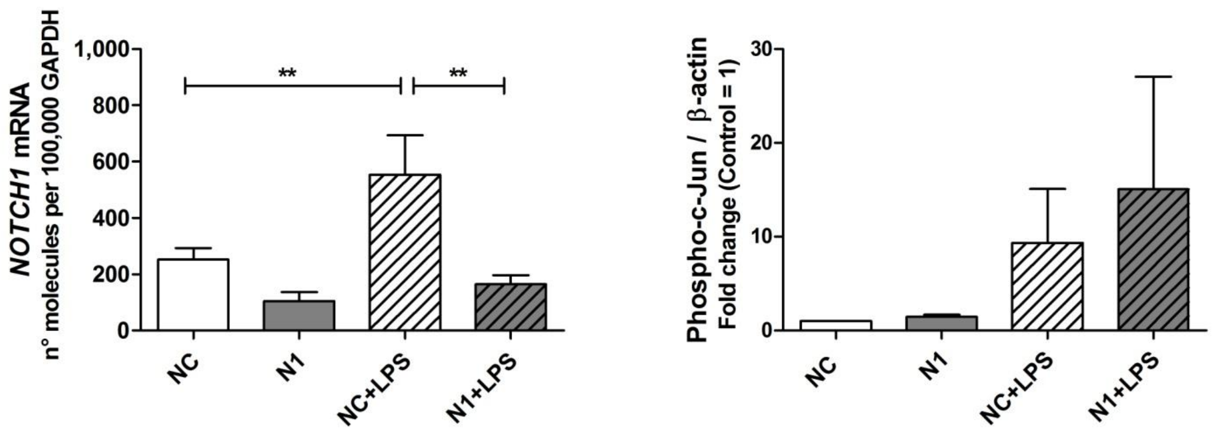

Supplementary Figure S2

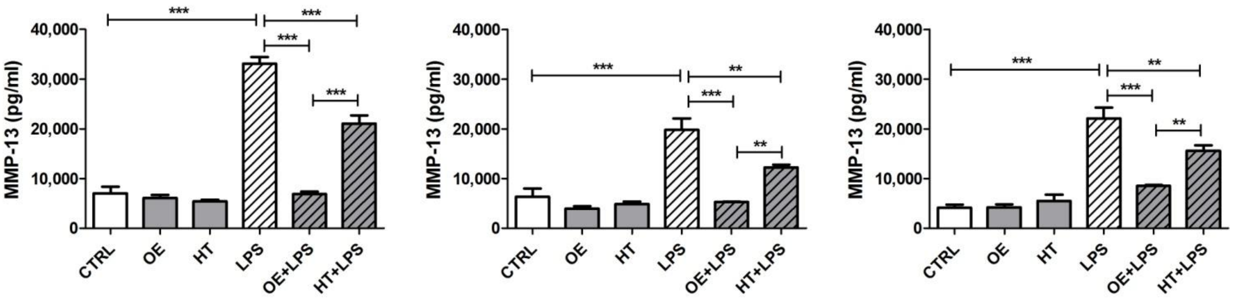

Supplementary Figure S3

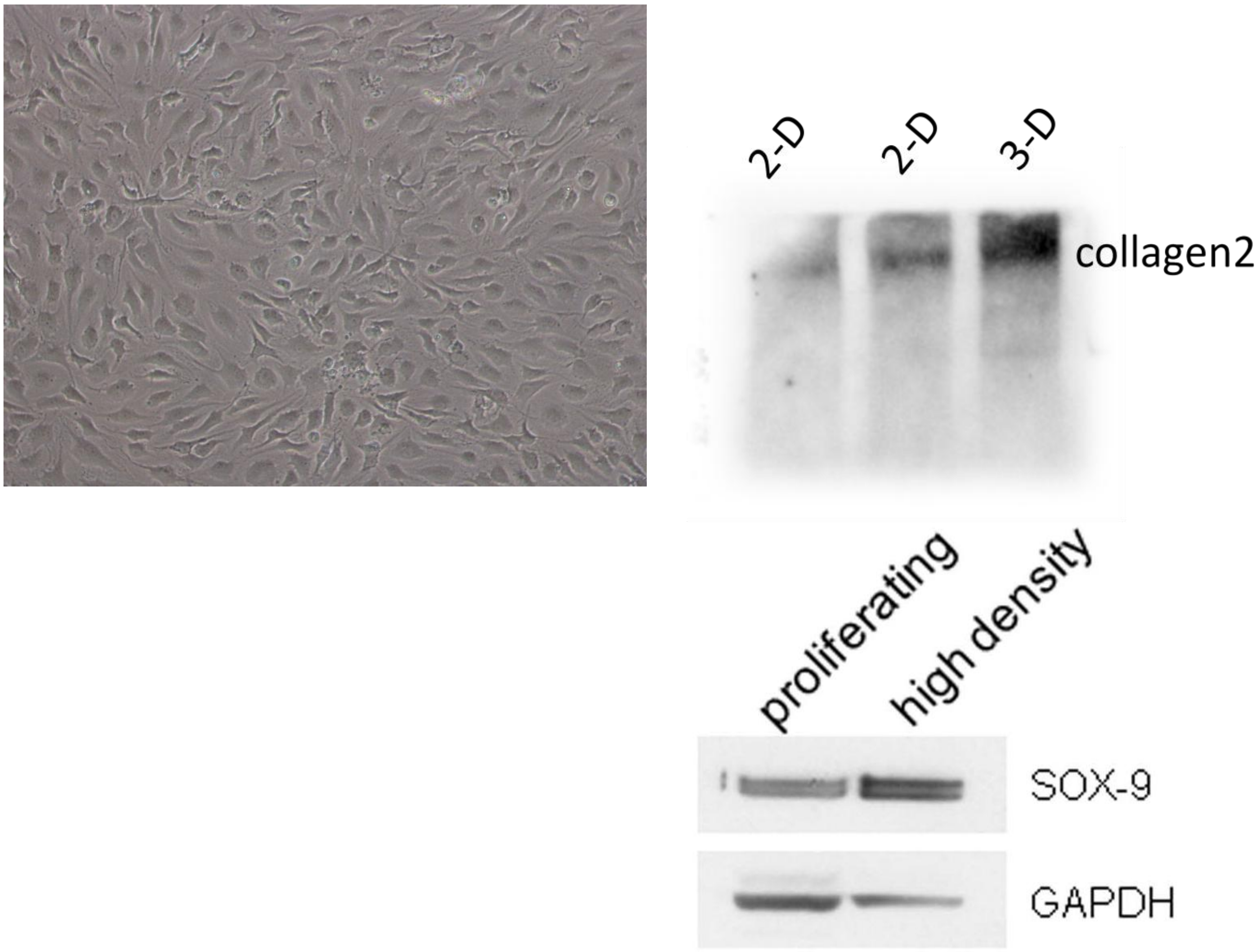

LEGEND TO SUPPLEMENTARY FIGURES

Supplementary Figure S1: NOTCH1 silencing mimics the effect of DAPT in increasing the levels of phospho c-Jun after LPS stimulation. Primary chondrocytes were treated with NOTCH1 siRNA (N1, ON-TARGETplus SMART pool with si-NOTCH1) or control siRNA (NC, treated with ON-TARGETplus Non-targeting Pool, as described in [1]). Left figure: efficiency of NOTCH1 silencing was confirmed by significant reduced NOTCH1 mRNA expression following LPS treatment (n=5). Right figure: in both unstimulated and LPS-stimulated conditions the level of activated (phosphorylated) c-Jun (normalized to β-actin) was increased in N1 compared to NC chondrocytes (n=3). Statistical analysis was performed by ANOVA, followed by Newman-Keuls' post hoc test, with $^{**}p < 0.01$.

Supplementary Figure S2: Inhibiting effects of OE and HT on LPS induced MMP-13 release from OA chondrocytes, following 24h exposure to LPS. No pattern: unstimulated samples; dashed pattern: LPS stimulated samples; white fill: no pretreatment; grey fill: nutraceutical pretreatment. Three different primary cultures were tested, with triplicate wells for each condition, so that each column represents the mean±SEM (n=3). Results obtained from the three different primary cultures were represented with the same Y scale to highlight the different inducibility. However, across the different conditions the trend was similar, with oleuropein always exhibiting a complete inhibiting activity on MMP-13 release induced by LPS treatment. Statistical analysis was performed by ANOVA, followed by Newman-Keuls' post hoc test, with $^{**}p < 0.01$, $^{***}p < 0.001$.

Supplementary Figure S3: Morphological and phenotypical characterization of primary chondrocytes used in the study. Left image is representative of the primary cultures used in our manuscript and shows that, as previously reported [2], when kept at high density, chondrocytes retain a round to polygonal instead of the elongated shape of dedifferentiated chondrocytes [2]. On the right: upper image shows the western blot assessment of collagen 2 expression in 2 lysates of chondrocytes kept at high density monolayer (2-D) and one lysate of chondrocytes kept in 3-D culture. The lysates were treated with pepsin as described in [2], subjected to SDS-PAGE and western blot as described in the main manuscript and probed with a 1:1000 dilution of anti-collagen 2 antibody (Chemicon MAB8887); lower image is derived from our previous paper [3] and compares the SOX9 level of proliferating versus high density culture (protein lysates derived from 100,000 cells, loaded in each well). This image confirms that the experimental settings used in the manuscript (high density cultures) are respectful of the differentiated phenotype with higher level of SOX9 compared to proliferating chondrocytes [3,4].

REFERENCES

1. Minguzzi, M.; Panichi, V.; D'Adamo, S.; Cetrullo, S.; Cattini, L.; Flamigni, F.; Mariani, E.; Borzi, R.M. Pleiotropic roles of notch1 signaling in the loss of maturational arrest of human osteoarthritic chondrocytes. *Int J Mol Sci* **2021**, *22*.
2. Otero, M.; Favero, M.; Dragomir, C.; Hachem, K.E.; Hashimoto, K.; Plumb, D.A.; Goldring, M.B. Human chondrocyte cultures as models of cartilage-specific gene regulation. *Methods Mol Biol* **2012**, *806*, 301-336.
3. Assirelli, E.; Pulsatelli, L.; Dolzani, P.; Platano, D.; Olivotto, E.; Filardo, G.; Trisolino, G.; Facchini, A.; Borzi, R.M.; Meliconi, R. Human osteoarthritic cartilage shows reduced in vivo expression of il-4, a chondroprotective cytokine that differentially modulates il-1beta-stimulated production of chemokines and matrix-degrading enzymes in vitro. *PLoS One* **2014**, *9*, e96925.
4. Ulivi, V.; Tutolo, G.; Mallein-Gerin, F.; Daga, A.; Cancedda, R.; Cancedda, F.D. A common pathway in differentiation and inflammation: P38 mediates expression of the acute phase sip24 iron binding lipocalin in chondrocytes. *J Cell Physiol* **2006**, *206*, 728-737.
